# *Trichomonas vaginalis* and Associated Factors among Pregnant Women Attending Antenatal Care at Bule Hora University Teaching Hospital, Oromia Region, Southern Ethiopia

**DOI:** 10.1155/2023/4913058

**Published:** 2023-12-14

**Authors:** Oliyad Husen, Alqeer Aliyo, Kalicha Boru, Tibeso Gemechu, Wako Dedecha, Girma Ashenafi

**Affiliations:** Department of Medical Laboratory Science, Institute of Health, Bule Hora University, Bule Hora, Ethiopia

## Abstract

Trichomoniasis is caused by a flagellated protozoan parasite called *Trichomonas vaginalis*. It is one of the most common, curable nonsexually transmitted infections globally. In Ethiopia, complications associated with genital infection in pregnant women are a common problem. Despite the burden of the disease, epidemiological data related to this disease is currently rare in Africa, particularly in Ethiopia. *Objective*. This research is aimed at assessing the prevalence of *Trichomonas vaginalis* and associated factors among pregnant women attending antenatal care at Bule Hora University Teaching Hospital. *Methods*. An institutional-based cross-sectional study was conducted among 196 pregnant women attending ANC at Bule Hora University Teaching Hospital. Structured questionnaires were used to collect sociodemographic and associated factor data. The consecutive sampling technique was used to include study participants. The two vaginal swabs were collected by brushing the vagina with a sterile cotton swab and tested by using direct wet mount and the Giemsa staining. The data were analyzed using SPSS version 26 for logistic regression analysis. A *p* value < 0.05 with 95% CI was used to declare it statistically significant. *Result*. An overall prevalence of *T. vaginalis* among pregnant women was 7.7% (95% with confidence interval (CI), 0.043-0.123). The highest prevalence was observed among the 35–39-year-old age group with 18.2% and among widowed women with 25%. This study revealed that the number of sexual partners (AOR: 3.215, 95% CI: 1.062-9.731) was a significant associated factor of *T. vaginalis*.*Conclusion*. The prevalence of *T. vaginalis* was considerably high among pregnant women in this study. This finding emphasizes the need for routine screening and treatment of pregnant women in the first antenatal care and enhances the need for regular health education for pregnant women at antenatal clinics to make them aware of their health, and avoidance of the risk of trichomoniasis is advised.

## 1. Introduction

*Trichomonas vaginalis* is a protozoan parasite that infects the human urogenital tract and is the causative agent of trichomoniasis [1]. It is an anaerobic flagellated parasite with a simple life cycle and involves only direct transmission of viable trophozoites [2, 3]. *T. vaginalis* infection in pregnancy has been shown to be related to serious pregnancy outcomes [4]. The most typical symptoms of trichomoniasis among women include foul-smelling vaginal discharge, vaginal irritation, lower abdominal pain, vulvovaginal secretion, and dysuria with serious sequelae such as preterm rupture of membranes, chorioamnionitis, preterm delivery, postabortal sepsis, ectopic pregnancy, tubal factor infertility, and low birth weight [5, 6]. The typical means of diagnosing trichomoniasis is a wet mount microscopic examination [7]. Metronidazole and tinidazole are first-line treatments for trichomoniasis [8].

Trichomoniasis is the most prevalent STI, with an estimated 156 million cases annually in 2016, accounting for almost half of the worldwide STI incidence this year [9]. Trichomoniasis is a disease with easy diagnosis and prevention; however, the prevalence remains high at global and national levels [10]. Its prevalence in pregnant women ranges from 17 to 20% in Africa, 16 to 53% in the USA, and 0.8% in Asia [11]. *T. vaginalis* infection is ten times more common in women than in men [12].

The documented data on the prevalence of *T. vaginalis* infection, particularly in pregnant women, is insufficient in Africa due to the lack of screening program [13]. Currently, there is no control program for trichomoniasis in Africa, so one can consider it a neglected infection by most health services [10, 14].

The high frequency of trichomoniasis among women is attributed to a number of variables, including poor personal cleanliness, having several sexual partners, and low socioeconomic level [15]. Additionally, *T. vaginalis* has been linked to an increased risk of HIV infection and cervical cancer [16]. Unexpectedly, the risk of vaginal trichomoniasis has increased, especially in emerging nations and among individuals who engage in high-risk behaviours such having many partners and engaging in heavy sexual activity [17]. Up to 44% of pregnant women are at risk for persistence or reinfection, according to earlier research [18].

The Ethiopian health policy follows the WHO recommendation, advocating the syndromic management of curable sexually transmitted disease infections and encouraging pregnant women for regular surveillance and screening for HIV and syphilis. Nevertheless, despite the high global incidence and high associated morbidity, curable STIs such as *T. vaginalis* have received comparatively little public health attention even in the area of research [15].

*T. vaginalis* is the most significant pathogen; despite this, to my knowledge, little is known about its epidemiology and risk factors in resource-limited countries such as Ethiopia. Therefore, this study is aimed at assessing the prevalence of *Trichomonas vaginalis* and associated factors among pregnant women attending antenatal care at Bule Hora University Teaching Hospital.

## 2. Methodology

### 2.1. Study Area, Design, and Period

The study was conducted at Bule Hora University Teaching Hospital, Oromia Region, Southern Ethiopia. Bule Hora town is 475 km from Addis Ababa, the capital city of Ethiopia. The town has 8 kebeles (the group of villages and lowest administrative unit in Ethiopia) [16], within which there is one governmental hospital, one health center, and 8 health posts. Bule Hora University Teaching Hospital (BHUTH) was established in 1990 E.C. The hospital is the largest in the West Guji Zone and provides healthcare services to over 1.3 million catchment population. It serves as a teaching, training, and clinical service center. The antenatal clinic of BHUTH serves an average of 30-40 pregnant women per day, and approximately 15 beds are available for prenatal and postnatal services in the hospital. The hospital has an average of 10 deliveries per day and 5-10 maternal deaths per year. An institutional-based cross-sectional study was conducted at Bule Hora University Teaching Hospital from June to August 30, 2022 [19].

### 2.2. Study Population and Selection Criteria

All pregnant women between the gestational periods of 35^th^ to 37^th^ weeks participated in the study, while pregnant women with a history of antibiotic use within two weeks before recruitment and emergency obstetric conditions who needed immediate intervention were excluded from the study.

### 2.3. Sample Size Determination

The sample size for this study was determined by using the single population ratio formula. When the *T. vaginalis* proportion (14.2%) was taken from a previous study conducted in the Gondar region, there was a margin error of 5%, a confidence interval (CI) of 95%, and a nonresponse rate of 5% [17].

(1) $$\begin{array}{c} n=\frac{{Z^{2}}_{\alpha /2}\;P\left(1-p\right)}{d^{2}}, \end{array}$$

where *n* represents the sample size, *Z* is the value corresponding to a 95% level of significance = 1.96, *P* is the proportion of prevalence of *T. vaginalis* in pregnant women = 14.2, *d* is the marginal error assumed to be 5%, *q* = (1 − *p*) = (1 − 0.142) = 0.858, and *n* = (1.96)^2^ (0.142) (0.858)/(0.05)^2^ = 187. Adding a 5% nonresponse rate, which is 9, the final sample size was 196.

### 2.4. Sampling Techniques

We used a consecutive sampling technique, and one hundred ninety-six (196) pregnant women at 35-37 weeks of gestation attending routine antenatal clinics at Bule Hora Teaching Hospital during the study period who fulfilled the inclusion criteria were enrolled.

### 2.5. Data Collection Methods

Two days of training was given to the data collectors (two experienced nurses and midwives) on the purpose of the study, study, participant recruitment, questionnaire, how to obtain informed consent, and processing by the principal investigator (PI). Each study participant gave their written informed consent after being informed of the study's goals and methods. After written informed consent was obtained from the study participants, sociodemographic and medical history data were collected using structured questionnaires in face-to-face interviews and were complemented with a medical record review.

Two sterile cotton swabs were used to gather two vaginal swabs, one after the other, by qualified midwives and nurses. The questionnaire was initially developed in English, translated to Afan Oromo/Amharic, and then translated back to English by another language specialist to ensure consistency. It was modified from earlier research of a similar nature. The sample was transported to the Microbiology Skills Teaching Laboratory of Bule Hora University, Department of Medical Laboratory Sciences, within 30 minutes using physiological saline in the ice pack.

### 2.6. Laboratory Analysis and Diagnosis

The wet saline smear and Giemsa staining were prepared from the first and second vaginal swabs, respectively. The wet saline smear was immediately performed using clean, grease-free microscopic slides covered with a coverslip and examined for motile *T. vaginalis* under a 10x objective lens for motile trichomonads, followed by confirmation with a 40x objective lens [18]. The Giemsa stain was used to confirm the negative result of the wet saline smear. The prepared smear was fixed by submersion in methanol for one minute and then allowed to dry before the Giemsa staining. After being stained with Giemsa dye (HiMedia Laboratories, India), it was scanned for *T. vaginalis* at 100x magnification using a phosphate buffer solution diluted 1 part to 19 parts at a pH of 7.2 for 10 minutes. Both the internal and external structures of the organism were clearly visualized in the specimen with the presence of *T. vaginalis* trophozoite [20].

### 2.7. Data Quality Control

Two days of training was given to the data collectors on the purpose of the study, study participant recruitment, the questionnaire, how to obtain informed consent, vaginal swab collection, and processing. Properly designed data collection tools and manuals were used. During sample collection, transportation, and processing steps, standard operating procedures (SOPs) were followed strictly.

The questionnaire was pretested on 5% of pregnant women sampled at Yabelo Hospital before the actual study, and necessary changes were made to the study tool. Data collectors were supervised by the principal investigator (PI), and data validity and completeness were checked by the PI.

### 2.8. Data Processing Analysis

Data analysis was performed using a computer with SPSS version 26 software. Frequency distribution was used to calculate prevalence in the overall study population and separately by associated risk factors. Descriptive statistics were computed to describe a relevant variable and expressed in the form of texts, tables, and graphs. Most of the variables were fitted to bivariable logistic regression. Bivariable logistic regression was used to compare the prevalence of *T. vaginalis* with each associated factor. An adjusted odds ratio (AOR) was used to determine the strength of the association. A variable with a *p* value < 0.25 within the bivariable analysis was further analyzed using multivariable logistic regression. A *p* value less than 0.05 with 95% CI was considered statistically significant.

## 3. Results

### 3.1. Sociodemographic Characteristics of Study Participants

A total of 196 pregnant women ranging in age from 17 to 37 years with a mean age of 25.97 years and a standard deviation of 4.69 years were enrolled. Greater than half of the responders (72, 36.7%) were between the ages of 25 and 29 years. The majority of the study participants (179, 91.3%) were married. In terms of residence, 124 (63.3%) were urban dwellers. Concerning educational status, 67 (34.2%) had no formal education, and the majority, 175 (89.3%), were unemployed in terms of occupation (Table 1).

### 3.2. Obstetric and Clinical Information

The majority of the study participants, 108 (55.1%), were multigravida, 149 (76.0%) had four or more ANC visits, 134 (68.4%) had one sexual partner, 141 (71.9%) had no history of contraceptive use, 185 (94.4%) had no history of antibiotic use, 176 (89.8%) had no history of abortion, 182 (92.9%) had no history of chronic illness, and 159 (81.1%) reported having no vaginal itching. In this study, approximately 192 (98.0%) of the respondents were HIV negative. Nearly half of the 103 (52.6%) study participants had vaginal discharge, and 36 (18.4%) experienced dysuria (Table 2).

### 3.3. Prevalence of *T. vaginalis* Infection among Pregnant Women Who Attended ANC Clinics

The overall prevalence of *T. vaginalis* among study participants was 15 (7.7%) (95% confidence interval (CI), 0.043-0.123). The highest prevalence of *T. vaginalis*, 2 of 11 (18.2%), was observed in the 35-39-year age group, and the lowest prevalence, 4 of 72 (5.6%), was observed in the 25- to 29-year age group. In terms of marital status, the prevalence of *T. vaginalis* infection was highest among widowed individuals, 1 of 4 (25%), and lowest among married responders, 12 of 179 (6.7%). The prevalence of *T. vaginalis* infection was higher in rural settings, 7 of 72 (9.7%), and in urban settings, 8 of 124 (6.5%). Concerning educational status, the prevalence of *T. vaginalis* infection was highest at 8 to 60 (13.3%) among those who attended just primary education and lowest at 1 to 27 (3.7%) among those who joined college and above. The highest prevalence of *T. vaginalis* infection was observed among those who were employed, 2 of 21 (9.5%), while the lowest prevalence was seen among those who were currently unemployed, 13 of 175 (7.4%). The infection rates of *T. vaginalis* were 4.5%, 14.5%, and 25% among pregnant women who were multigravida, had just two or more sexual partners, and were HIV positive, respectively. The details are described in Table 3.

### 3.4. Factors Associated with the Prevalence of *T. vaginalis* Infection

A logistic regression analysis showed that only two variables were significantly connected with the prevalence of *T. vaginalis* infection among those variables that were candidates for multivariable analysis. The results of the current study indicated that pregnant women who had two or more sex partners were three times (AOR: 3.094, 95% CI: 1.003-9.549, *p* = 0.049) more likely to contract *T. vaginalis* than women who had just one sex partner (Table 3).

## 4. Discussion

In the present study, the overall prevalence of *T. vaginalis* among pregnant women who attended ANC services was 7.7% (95% confidence interval (CI), 0.043-0.123). This finding was in line with the findings reported from Ethiopia (4.8-5.3%) [21, 22], Kenya (6.9%) [23], Ghana (5-7.2%) [24], Nigeria (8.1-10%) [25], South America (9%) [10], Iran (5.64%) [26], and Asia (7.1%) [27]. However, the current finding was higher than the study reported from Southern Ethiopia (3.1%) [28], Ghana (1.7%) [24], Benin (2.8%) [29], South Africa (1.2%) [30], and Portugal (1.0%) [31] compared to the current study. The possible reason for this high prevalence among study participants might be due to variations in geographic location, many numbers of sexual partners, low personal hygiene status as many participants have no formal educational level, the association of HIV with trichomoniasis, and behavioural factors.

Moreover, this finding was lower than that in studies conducted in Nigeria (12.5%) [32], South Africa (20%) [30], and Yemen (11.1%) [33]. This might be due to differences in detection methods, sample size variation, and geographical location.

Of the independent variables that were assessed, the number of sexual partners was significantly associated with the prevalence of *T. vaginalis* infection. The number of sexual partners was an independent factor significantly associated with the prevalence of *T. vaginalis,* which was in agreement with studies conducted in Ethiopia [34], Nigeria [25], and Brazil [10]. The increasing number of sexual partners is one factor that increases the infection rate of *T. vaginalis* [35].

### 4.1. Limitations of the Study

This study is an institutional-based cross-sectional study and includes only pregnant women who were attending ANC service at Bule Hora University Teaching Hospital, which might not represent the general population. In addition, only direct wet mount microscopy and the Giemsa staining were performed for *T. vaginalis* identification, which is less sensitive than culture, PCR, and ELISA.

## 5. Conclusion and Recommendations

In the present study, the overall prevalence of *T. vaginalis* was 7.7% among pregnant women attending antenatal care at Bule Hora University Teaching Hospital. It was also noted that there was a significant association between *T. vaginalis* infection and the number of sexual partners. A general awareness campaign and ongoing health education for pregnant mothers attending antenatal clinics were advised to teach them about their health, prevent hazardous behaviours, and address the risk of *T. vaginalis* infection during their pregnancy.

## Figures and Tables

**Table 1:** Sociodemographic features of pregnant women following antenatal care at Bule Hora University Teaching Hospital, Southern Ethiopia, 2022 (*n* = 196).

Variables	Categories	Frequency (*n*)	Percentage (%)
Age	15-19	19	9.7
	20-24	51	26.0
	25-29	72	36.7
	30-34	43	21.9
	35-39	11	5.6

Marital status	Single	7	3.6
	Married	179	91.3
	Divorced	6	3.1
	Widowed	4	2.0

Residency	Urban	124	63.3
	Rural	72	36.7

Educational level	No formal education	67	34.2
	Grades 1-8	60	30.6
	Grades 9-12	42	21.4
	College and above	27	13.8

Occupation	Employed	21	10.7
	Unemployed	175	89.3

Abbreviation: HIV: human immunodeficiency virus.

**Table 2:** Obstetric and clinical information of pregnant women following antenatal care at Bule Hora University Teaching Hospital, Southern Ethiopia, 2022 (*n* = 196).

Variables	Categories	Frequency (*n*)	Percentage (%)
Gravidity	Primigravida	88	44.9
	Multigravida	108	55.1
ANC visit	<4	47	24.0
	≥4	149	76.0
History of contraceptive use	Yes	55	28.1
	No	141	71.9
History of antibiotic use	Yes	11	5.6
	No	185	94.4
Number of sex partner	One	134	68.4
	≥2	62	31.6
HIV status of mother	Positive	4	2.0
	Negative	192	98.0
History of abortion	Yes	20	10.2
	No	176	89.8
Vaginal discharges	Yes	103	52.6
	No	93	47.4
History of chronic illness	Yes	14	7.1
	No	182	92.9
History of chronic illness	Yes	36	18.4
	No	160	81.6
Pain during urination	Yes	36	18.4
	No	160	81.6

**Table 3:** Bivariable and multivariable analysis of factors associated with T. vaginalis infection among pregnant women who followed ANC at Bule Hora University Teaching Hospital, Southern Ethiopia, 2022.

Variables	Category	TV (+), *N* (%)	TV (-), *N* (%)	COR (95% CI)	*p* value	AOR (95% CI)	*p* value
Age group	15-19	2 (10.5%)	17 (89.5%)	0.53 (0.06-4.41)	0.557		
	20-24	4 (7.8%)	47 (94.4%)	0.38 (0.06-2.41)	0.307		
	25-29	4 (5.6%)	68 (93.0%)	0.27 (0.04-1.66)	0.156^∗^	0.38 (0.05-2.95)	0.35
	30-34	3 (18.2%)	40 (81.8%)	0.33 (0.05-2.33)	0.270		
	35-39	2 (7.0%)	9 (92.3%)	1			

Marital status	Single	1 (14.3%)	6 (85.7%)	1	1		
	Married	12 (6.7%)	167 (93.3%)	0.43 (0.048-3.88)	0.453		
	Divorced	1 (16.7%)	5 (83.3%)	1.20 (0.06-24.47)	0.906		
	Widowed	1 (25.0%)	3 (75.0%)	2.00 (0.09-44.35)	0.661		

Residency	Urban	8 (6.5%)	116 (93.5%)	1			
	Rural	7 (9.7%)	65 (90.3%)	1.56 (0.54-4.50)	0.409		

Educational level	No formal education	4 (6.0%)	63 (94.0%)	1.65 (0.18-15.48)	0.661		
	Grades 1-8	8 (13.3%)	52 (86.7%)	4.00 (0.48-33.71)	0.202^∗^	2.81 (0.31-25.29)	0.36
	Grades 9-12	2 (4.8%)	40 (95.2%)	1.30 (0.11-15.08)	0.834	1	
	College and above	1 (3.7%)	26 (96.3%)	1			

Occupation	Employed	2 (9.5%)	19 (90.5%)	1			
	Unemployed	13 (7.4%)	162 (92.6%)	0.76 (0.16-3.64)	0.734		

Gravidity	Primigravida	4 (4.5%)	84 (95.5%)	1		1	
	Multigravida	11 (10.2%)	97 (89.8%)	2.38 (0.73-7.76)	0.150^∗^	3.08 (0.88-10.69)	0.28

ANC visit	<4	3 (6.4%)	44 (93.6%)	1			
	≥4	12 (8.1%)	137 (91.9%)	0.78 (0.21-2.89)	0.708		

History of contraceptive use	Yes	4 (7.3%)	51 (92.7%)	0.93 (0.28-3.05)	0.900		
	No	11 (7.8%)	130 (92.2%)	1			

History of antibiotic use	Yes	1 (9.1%)	10 (90.9%)	1.22 (0.15-10.24)	0.854		
	No	14 (7.6%)	171 (92.4%)	1			

Number of sex partner	1	6 (4.5%)	128 (95.5%)	1		1	
	≥2	9(14.5%)	53 (85.5%)	0.28 (0.09-0.81)	0.020^∗^	3.09 (1.00-9.55)	0.049^∗∗^

HIV status	Positive	1 (25.0%)	3 (75.0%)	4.24 (0.41-43.46)	0.224^∗^	1.96 (0.07-55.16)	0.693
	Negative	14 (7.3%)	178 (92.7%)	1		1	

History of abortion	Yes	2 (10.0%)	18 (90.0%)	1.393 (0.29-6.67)	0.678		
	No	13 (7.4%)	163 (92.6%)	1			

Vaginal discharges	Yes	10 (9.7%)	93 (90.3%)	1.89 (0.62-5.76)	0.261		
	No	5 (5.4%)	88 (94.6%)	1			

History of chronic illness	Yes	2 (14.3%)	12 (85.7%)	2.17 (0.44-10.73)	0.343		
	No	13 (7.1%)	169 (92.9%)	1			

Dysuria	Yes	4 (11.1%)	32 (88.9%)	1.69 (0.51-5.66)	0.392		
	No	11 (6.9%)	149 (93.1%)	1			

Abbreviations: TV (+): *Trichomonas vaginalis* positive; TV (-): Trichomonas vaginalis negative; HIV: human immunodeficiency virus; ANC: antenatal care; COR: crude odds ratio; AOR: adjusted odds ratio; CI: confidence interval. Note: ^∗^ indicates the variable with *p* < 0.25 in the bivariable analysis, and ^∗∗^ indicates the variable with *p* < 0.05 in the multivariable analysis and is statistically significant.

## Data Availability

Data for this research are available and included in this manuscript. Therefore, we can communicate you when you need for future process.

## Abbreviations

ANC: Antenatal care
BHUTH: Bule Hora University Teaching Hospital
ELISA: Enzyme-linked immunosorbent assay
HIV: Human immunodeficiency virus
SOP: Standard operating procedure
STI: Sexually transmitted infection
UTI: Urinary tract infection
WHO: World Health Organization.

## Supplementary Materials

Supplementary Materials Questionnaire and laboratory procedures.
