# Supplementary material for: Trichomonas vaginalis and Associated Factors among Pregnant Women Attending Antenatal Care at Bule Hora University Teaching Hospital, Oromia Region, Southern Ethiopia
Source: J Parasitol Res. 2023 Dec 14;2023:4913058. doi: 10.1155/2023/4913058 (PMC10735719; doi:10.1155/2023/4913058)
Supplement: Supplementary Materials — Questionnaire and laboratory procedures. [file 4913058.f1.docx]

**Appendix-III: English version of the questionaries**

| Questionaries for the prevalences of *Trichomonas vaginalis* and associated risk factors, and among pregnant women attending antenatal care at Bule Hora University Teaching Hospital  Patient ID ____________________  Date of interview and data collection (date/month/year): ________/ _____/_________  Interviewer’s name & signature: ____________________  Supervisor’s name & signature: ______________________  Participant Name______________________________________ | | |
| --- | --- | --- |
| **Codes** | **Socio-demographic factors** | |
| 000 | Age | _____ years/months |
| 001 | Marital status | Single  Married  Divorced  Widowed |
| 002 | Residency | Urban  Rural |
| 003 | Educational status | No formal education  Grade 1-8  Grade 9-12  College and University |
| 004 | Occupation | Privately employed  Government worker  House wife  Student  Other |
| **Codes** | **Behavioral factors and Infection prevention practices** | |
| 005 | Gravidity | Primigravid  Multigravid |
| 006 | Number of ANC visits | _____________ |
| 007 | Life time number of sex partner | One  Two and above |
| 008 | History of contraceptive use | Yes  No |
| 009 | History of antibiotics use | Yes  No |
| **Codes** | **Clinical factors** | |
| 010 | HIV status of mother | Yes  No |
| 011 | History of stillbirth | Yes  No |
| 012 | History of abortion | Yes  No |
| 013 | Vaginal itching | Yes  No |
| 014 | Vaginal discharges | Yes  No |
| 015 | History of preterm delivery | Yes  No |
| 016 | History of Premature rupture of membrane | Yes  No |
| 017 | History of chronic illness | Yes  No |
| 018 | Pain during urination (dysuria) | Yes  No |

**Appendix IV: Laboratory procedures**

**Laboratory protocol**

- Provide written consent form for pregnant women willing to participate in the study prior to sample collection
- Collection of clinical data from physician/midwife
- Socio-demographic data will be collected with well standardized questionnaire

**Specimen collection**

1. The patient is positioned in lithotomy position on the exam table (as for a pelvic examination).

2. Using sterile rayon tipped swabs to obtain a sample of vaginal discharge. The fluid specimen can be obtained directly from the mucosa of the posterior vagina.

**Laboratory Procedures for *T. vaginalis***

- 1. **Direct wet mount**

It is used for the detection of trophozoites stages of *T. vaginalis* under microscopes

**Reagents and equipment**:

Aqueous sodium chloride, glass microscope slide, and coverslip, 0.5ml physiological sterilized saline/Amies transport media.

**Procedures**

1. Place a drop of saline onto the middle of the glass slide. (Alternative method: Place several drops of saline in a small glass test tube and place the swab in the tube. The swab can then be wiped onto a slide at a later time.)
2. Mix a small amount of vaginal fluid to be examined into the saline drop.
3. Overlay a coverslip.
4. Examine directly through microscope at ×40 and ×100 (oil immersion)

**Interpretation**

Saline wet mount demonstrating oval-bodied, flagellated trichomonads. They are similar in size to leukocytes and can be distinguished from them by their motility and presence of flagella
